# Investigation on Ultrasonic Welding Attributes of Novel Carbon/Elium^®^ Composites

**DOI:** 10.3390/ma13051117

**Published:** 2020-03-03

**Authors:** Somen K. Bhudolia, Goram Gohel, Kah Fai Leong, Robert J. Barsotti

**Affiliations:** 1 School of Mechanical and Aerospace Engineering, Nanyang Technological University, 50, Nanyang Avenue, Singapore 639798, Singapore; goram001@e.ntu.edu.sg (G.G.); MKFLEONG@ntu.edu.sg (K.F.L.); 2 Institute for Sports Research, Nanyang Technological University, 50, Nanyang Avenue, Singapore 639798, Singapore; 3 Arkema Pte Ltd, Singapore 117528, Singapore; robert.barsotti@arkema.com

**Keywords:** polymer–matrix composites (PMCs), thermoplastic resin, ultrasonics, joints/joining

## Abstract

Joining large and complex polymer–matrix composite structures is becoming increasingly important in industries such as automobiles, aerospace, sports, wind turbines, and others. Ultrasonic welding is an ultra-fast joining process and also provides excellent joint quality as a cost-effective alternative to other joining processes. This research aims at investigating the welding characteristics of novel methyl methacrylate Elium^®^, a liquid thermoplastic resin. Elium^®^ is the first of its kind of thermoplastic resin, which is curable at room temperature and is suitable for mass production processes. The welding characteristics of Elium^®^ composites were investigated by optimizing the welding parameters with specially designed integrated energy directors (ED) and manufactured using the Resin transfer molding process. The results showed a 23% higher lap shear strength for ultrasonically welded composite joints when compared to the adhesively bonded joints. The optimized welding time for the ultrasonic welded joint was found to be 1.5 s whereas it was 10 min for the adhesively bonded joint. Fractographic analysis showed the significant plastic deformation and shear cusps formation on the fractured surface, which are typical characteristics for strong interfacial bonding.

## 1. Introduction

Thermoplastic (TP) composites are preferred due to their excellent damping, impact, fracture toughness, recyclability properties and their ability to be fused or welded to itself or with other materials. Thermoplastic resin has an inherent ability to soften once heated above the defined temperature range and retain their properties once they are cooled down. Hence, the manufactured TP composite is an attractive candidate for welding to TP composites and also with dissimilar materials like thermoset (TS) composites and metals. There is growing call from a wide spectrum of industries (aerospace, automotive, sports, and many more) to eradicate the classical ways of joining the polymer composite parts viz. mechanical fastening and the usage of control adhesives. The major drawback of using the former is that composites are susceptible to high-stress concentration generated due to the holes and its labor intensiveness whilst the latter requires an incredibly longer curing time as well as the longer surface preparation [1,2]. Both of the conventional approaches of joining hinders the realistic chances of achieving shorter production cycles and are not suitable for automation processes. Welding attributes of thermoplastics aids in the cost-effectiveness of the composite part to be manufactured in an industrial environment from forming until the finishing steps [3,4,5,6]. The most feasible welding techniques available for fusion bonding of thermoplastic composites are resistance [7,8,9], induction [8,10,11,12,13,14,15] and ultrasonic welding [6,16,17,18,19,20,21,22,23,24,25,26,27,28,29,30,31,32,33,34,35]. These behave differently in the way the heat is generated at the welding interface. Ultrasonic welding is an ultrafast process of joining thermoplastic composites and works on the principle of the application of high frequency and low amplitude vibration at the interface of the joining surfaces of the adherends to be welded.

Many researchers have carried out extensive studies on the welding attributes of different types of thermoplastic composites [36]. Liu et al. [20,37] investigated the effect of different welding parameters like weld time, weld pressure, the geometry of the energy director (ED), amplitude, hold time, and hold pressure on the weld quality using the Taguchi method. The material used for the study was polypropylene (PP) reinforced glass fiber composites and Nylon 6 reinforced glass fiber composites. Both investigations showed that weld time, the amplitude of vibration, and ED geometry had a significant effect on the weld quality. The energy director also has a significant effect on the weld quality as it allows for energy concentration during the joining process [24,38,39]. Y.K. Chuah et al. investigated the effect of ED by ultrasonically welding pure acrylonitrile butadiene styrene (ABS) and a polyethylene (PE) thermoplastic with different energy director configurations viz. semi-circular, triangular, and rectangular [39]. The semi-circular shape was found to be the most efficient welding condition while the triangular ED showed the lowest result. A similar study was carried out by Villegas on the effect of the weld quality by using different configurations, direction, and shape of the energy director [24]. A polyetherimide matrix reinforced carbon fiber composite was used for the investigation and the results were examined by carrying out a static lap shear test. Results showed that using multiple ED allowed for maximum coverage of the overlap area, hence also provides the maximum strength.

An energy control mode was used by Keita Goto et al. [34] and constant time mode was used by Wang Tao et al [35] to investigate the welding efficiency using the flat ED. Wang Tao et al. investigated the effect of different welding times on the welding strength of CF/PEEK composites using a flat ED with a 0.45 mm thickness [35]. Results from this study showed that with the gradual increase in time, the weld strength also increased. In contrast, after an optimum time, a further increase in time resulted in larger cracks and voids and the weld strength was significantly reduced. Aerospace, automotive industries, wind turbines, and other industries require lightweight material to increase efficiency by reducing energy consumption. Hence, using a fastener or bolted joint will add the weight. Due to the long curing cycle of adhesive joints, ultrasonic welding is the preferred option for mass production in industries [40]. Palardy et al. [41] investigated and showed that ultrasonic welding could be scaled up by sequential welding (i.e., a continuous line of spot welding) will serve the same effect of a continuous weld.

A research study was carried out on the fusion bonding of the Elium^®^ composite by Murray et al. [42]. In this research, resistance welding with different heating elements and induction welding techniques were used to weld the Glass fiber Elium^®^ composites for wind turbine blade applications. There was around a 30% improvement in lap shear test for the welded samples compared to the one bonded with adhesives. At 10 million cycles (defined stress for no failure), the fatigue limit for a fusion-welded sample was found to be 5 MPa compared to 3 MPa in the case of the adhesively bonded sample. A preliminary study was also carried out by Bhudolia et al. to demonstrate the fatigue response of an ultrasonically welded Carbon/Elium^®^ composite [43]. The results showed a 7–12% higher fatigue strength of welded joints with integrated ED compared to adhesively bonded samples.

Recently, a novel acrylic thermoplastic resin, Elium^®^ has been developed by Arkema, which is a first of its kind TP resin system to cure at room temperature and possesses the same in-plane mechanical properties compared to the high-performance epoxy resin. It can be manufactured using liquid injection processes like Resin Transfer Molding (RTM) and vacuum infusion processes as it possesses a viscosity that can go as low as 50 cP. Recently, significant research has been reported in the literature investigating the impact [44,45,46,47,48], fracture toughness [49,50,51], vibration [52], flexure [53,54,55], tensile [55,56], and other mechanical attributes of this novel resin system with different fiber reinforcements. Current research aims at investigating the welding attributes of this novel resin system, which could pave an excellent way of joining Elium^®^ composite parts. It could be of significant importance for wind turbines, automotive, sporting, and other applications where the parts are currently joined using long cure and sophisticated control adhesives.

## 2. Materials and Manufacturing

### 2.1. Materials

For the manufacturing of thermoplastic composite laminates, FOE sized 12K 2 × 2 twill weave dry carbon fibers supplied from CHOMARAT were used as the reinforcement of 380 gsm [57] and Elium^®^ 150 thermoplastic resin was used (Arkema, France) as the matrix material. Elium^®^ 150 resin undergoes radical polymerization to form high molecular weight acrylic co-polymers with the addition of a benzoyl peroxide initiator at a mixture ratio of resin to hardener 100:3 at room temperature (RT) [4,46,49,58]. For the adhesive bonding study of composite materials, SAF 30 5 adhesives were used, provided by Bostik [59]. The important properties of the Elium^®^ 150 resin and the bonding properties for SAF 30 5 are shown in Table 1.

### 2.2. Manufacturing of Composite Laminates

#### 2.2.1. Manufacturing of Flat Composite Laminates

**Figure 1:**
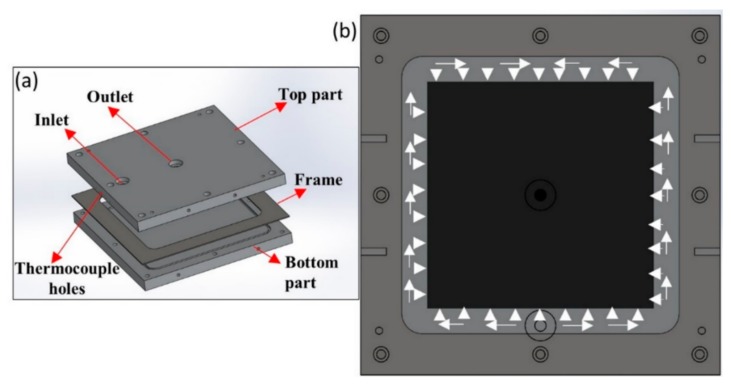
(**a**) Mold design for flat composite laminate manufacturing; (**b**) circumferential resin strategy.

To manufacture a flat composite laminate, a 3-part (top, bottom, and a frame) RTM mold was used as shown in Figure 1a with a frame thickness of 2 mm. Mold surface preparation was carried out with a release agent to ease the de-molding after manufacturing. Five layers of twill weave carbon fibers of 270 × 270 mm^2^ were used in order to achieve the fiber volume fraction of 54%. Each layer of the fibers was bonded using a binder and a heat gun was used to activate the binder. All of the layers were placed into the mold along with the frame and the top mold was closed. Elium^®^ resin was prepared by mixing it with benzoyl peroxide at a weight ratio of 100:3 and was injected into the mold at an inlet pressure of 2 bar and the outlet was set at atmospheric pressure in room temperature condition.

Figure 1b shows the flow trend of the resin. Resin flows circumferentially in the mold and is then introduced into the dry fibers. Once the part is completely filled and the excess resin comes out through the outlet, both inlet and outlet ports were clamped, and the laminate was allowed to cure at room temperature. Post-curing of the laminate was performed in the heat plate at 65 °C temperature for 45 min and later cooled to room temperature before demolding.

**Figure 2:**
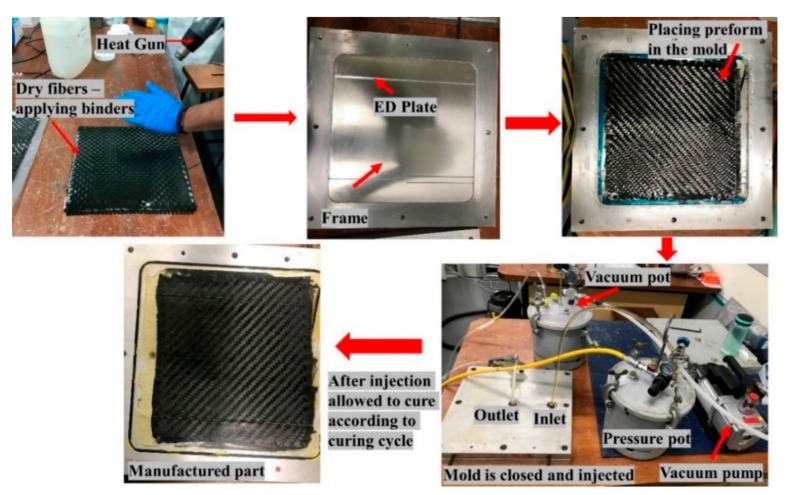
Manufacturing steps for the Resin Transfer Moulding (RTM) process to manufacture composite laminate.

Figure 2 refers to the general steps for the manufacturing of the composite laminate using the RTM manufacturing technique in the current research.

#### 2.2.2. Manufacturing of Energy Director (ED) Integrated Laminates

For the welding study, thermoplastic composite laminates were also manufactured with the integrated ED of semi-circular configuration, as recommended by many researchers in the literature [24,34,60]. The below subsections will give further details regarding the entire manufacturing process.

**Figure 3:**
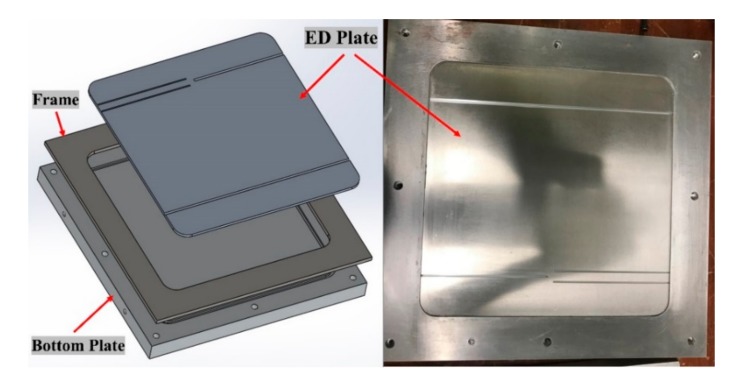
Mold design of the Energy Director (ED) composite laminate manufacturing.

**Mould Design**:

A four-part (top mold, bottom mold, frame, and ED plate) mold was conceptualized for use in the manufacturing of an ED integrated composite laminate. The ED integrated laminates that were manufactured were also of 2 mm thickness. A frame of 6 mm thickness was used wherein a 4 mm ED mold plate with semi-circular grove (refer Figure 3) was tight fitted to give the laminate a final thickness of 2 mm. The mold design of the ED integrated composite laminate manufacturing is shown in Figure 3.

**Figure 4:**
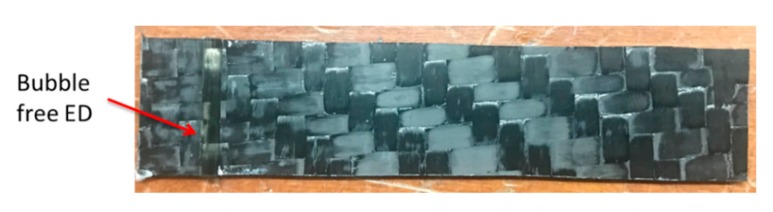
Manufactured Energy Director (ED) laminate with optimized parameters.

For the manufacturing of the ED integrated thermoplastic laminate, a similar method, as in the case of manufacturing flat laminates was used (refer Figure 2). After mold preparation, the preform was placed on the ED grooved plates so that when the resin is injected, it will flow into the grove and become cured. The final consolidated part will have a neat cured resin ED, acting as a concentrated energy source during the welding process. The injection parameters were different, which were obtained by different manufacturing trials and the optimization study. The optimized injection strategy was to keep the outlet at the vacuum of −0.91 bars and the resin was injected at an inlet pressure of 2 bars. This resulted in a clean and almost bubble-free ED integrated panel. It should be noted that it was also mandatory to apply the full vacuum before the resin injection to remove the entrapped air from the mold. Figure 4 shows the final bubble-free integrated ED laminate for welding.

## 3. Experiments

### 3.1. Welding Methodology

**Figure 5:**
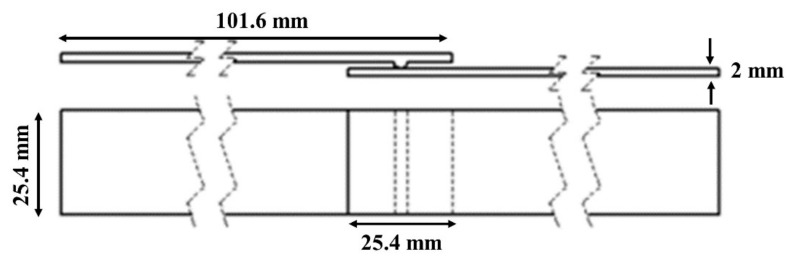
Schematic of Semi-circular ED Elium^®^ composite_ Flat Elium^®^ Composite (SC-ELC_FL-ELC) welding configuration.

**Figure 6:**
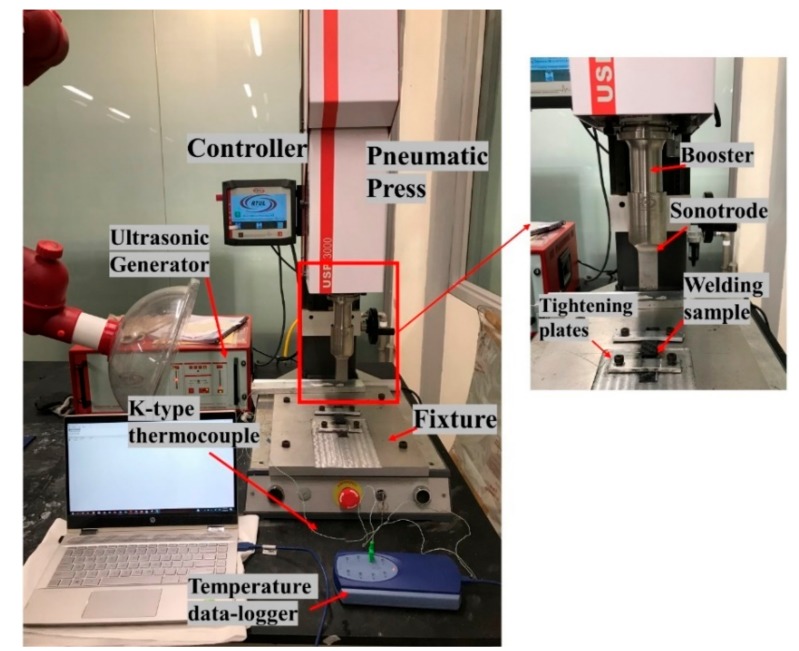
Ultrasonic welding machine test setup.

The standard lap-shear specimens [61] were manufactured to carry out the weld strength study (Figure 5). An ultrasonic welding machine with a maximum power output of 3000 W, generator of 20 kHz frequency, and AWC-6 microprocessor controller was used to weld the specimens in the current research as shown in Figure 6. To weld the laminates, a constant time mode was used and in the constant time mode, four parameters need to be set welding: weld time, hold time, weld pressure, and the amplitude. At 100% of the amplitude, the value transferred on to the surface of the adherend through the sonotrode was 65 µm.

Figure 6 shows the ultrasonic welding machine with the fixture designed to weld the lap joint samples. A step of 2 mm was milled to balance the offset with a specimen thickness of 2 mm. After placing the specimen into the fixture, it was fixed by the tightening mechanism to ensure that it did not move during the welding process. The SC-ELC_FL-ELC (Semi-circular ED Elium^®^ composite_ Flat Elium^®^ Composite) welding configuration was used in the current research. Note that the semi-circular ED Elium^®^ composite was kept on the top during welding. The schematic of the welding configuration is shown in Figure 5.

### 3.2. Adhesive Sample Preparation

Adhesive and hardener were mixed properly and then applied to the adherend surfaces. The mixed adhesive was applied to both the adherends in the required area and bound together. The adhesive thickness was found to be around 0.65 mm.

### 3.3. Lap Shear Test

A standard Lap shear test was performed to evaluate the static shear strength of the bonded joints due to its ease of usage as well as excellent reproducibility in the results [62]. The lap shear test was performed according to the ASTM D5868-01 standard [61]. A universal testing machine of 50 kN load capacity INSTRON was used for performing this test and the test was performed with a crosshead speed of 13 mm/min and was carried out at ambient conditions. Two different lap shear strengths (LSS) were calculated as suggested by Villegas et. al [24] due to a difference in the welded area, LSS1 and LSS2. LSS1 ids calculated as the peak load divided by the total overlap area and LSS2 is calculated as the peak load divided by the effective welded area. Thus, LSS1 defines the effectiveness of the joint and LSS2 defines the weld quality. The effective welded area was calculated by observing the actual welded area of the fracture surface after the specimen was statically tested with ImageJ software (1.8.0_112). Table 2 depicts the technical specification used for lap shear testing of the specimens.

### 3.4. Fractographic Investigation

The microscopic investigation was carried out on the fractured welded samples under static lap shear tests to check the failure mechanisms. A stereotype optical microscope (OLYMPUS S Z X7) was used to check the quality of the weld. Scanning electron microscope (SEM), JEOL SEM 5 600 LV was used to carry out the fractographic investigation and to check the associated failure mechanisms on the top and bottom surfaces of the adherends.

## 4. Results and Discussions

### 4.1. Initial Welding Trials

Initially, all the welding trials were carried out at 100% amplitude with varying weld time and weld pressure. However, the weld resulted in (1) excess matrix flowing out of the interface and (2) massive delamination of the top adherend. Samples were also welded at the lower weld time and lower pressure at 100% amplitude, but the same phenomenon was observed. This can be attributed to the higher energy transfer at the weld interface. Therefore, the amplitude was further reduced to 75% and the welded samples were statically tested in lap shear. It showed more promising results in lap shear tests compared to the one with 100% weld condition. Additionally, from the literature, it is advised to weld the acrylic-based matrix in the range of 40–70 µm amplitude [63]. The maximum amplitude in the current welding machine is 65 µm and 75% of this amplitude (48.75 µm) falls within the desirable limit of the ultrasonic welding for acrylic polymers. Few samples were welded with a 50% amplitude, but the energy director was not melted and a visible gap between the adherends was observed. In most of the available research [34,35], the amplitude is kept constant and other parameters are varied, so in the current investigation, for the ED integrated samples, 75% (48.75 µm) amplitude was fixed in the welding optimization study.

**Figure 7:**
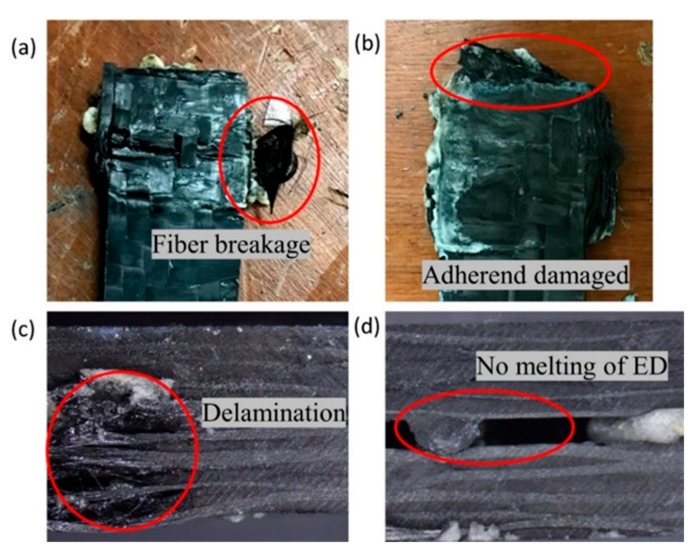
Visual and microscopic pictures of initial welding trials (**a**–**c**) over-bonded (**d**) un-bonded joints.

**Figure 8:**
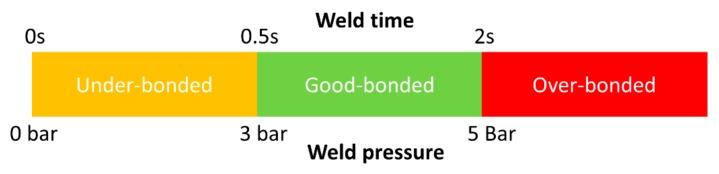
Effect of near and far-field weld parameters on the bonding of ED integrated panels.

In order to fix the weld time range, initially, the samples were welded in the time range from 0.5 s to 3 s and the joint strength was evaluated visually and by the lap shear test. At a lower time of 0.5 s and high-pressure of 4–5 bars, satisfactory results were achieved, but when samples were welded at higher welding times above 2 s, it resulted in significant damage to the adherend. At higher weld time and even at lower pressure, the top adherend was damaged by delamination and fiber crushing. The adherend failed by minimal manual force (pulling by hand) as it was over-bonded and damaged. Thus, the range of welding time for the current research was selected to be from 0.5s to 2 s with a 0.5 s interval. Considering hold time is not a significant weld parameter [35,37], so in the current investigation, it was kept constant at 2 s. Weld pressures selected for the current research were 3, 4, and 5 bar. Weld pressure of higher than the 5 bar showed delamination of the upper adherend as noted in the case with a higher weld time and amplitude. While at a pressure lower than 2 bar, the bond was very weak where it could be fractured with manual force. In the ultrasonic welding, the energy is transferred to the interface from the top adherend, hence in the current study, the semi-circular ED Elium^®^ adherend was kept on the top. Figure 7 shows the under welded and over bonded specimen during the initial trials. Figure 8 shows a summary of the initial trials and the effect of the welding parameters (weld time and weld pressure) on the bonding conditions.

### 4.2. Design of Experiments

In the current research, full factorial design (FFD) was used for the design of the experiments [64]. From the initial trails above-mentioned, the welding parameters selected for the welding of the adherends are shown in Table 3.

### 4.3. Adhesively Bonded Joints

**Figure 9:**
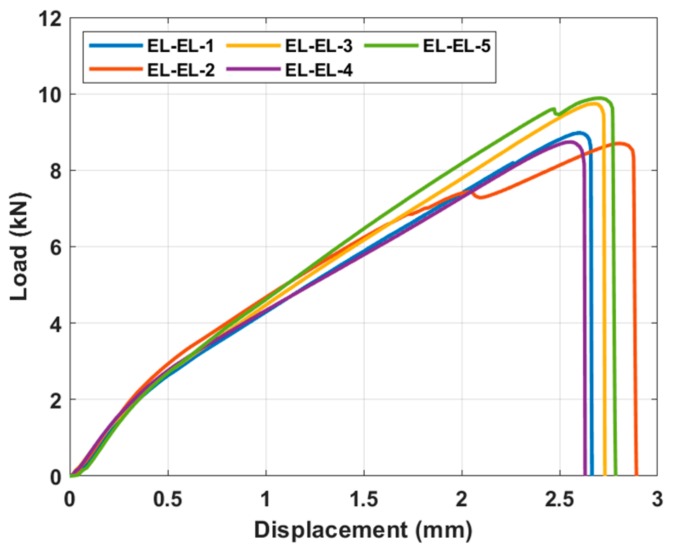
Load vs. displacement curves for the adhesively bonded Elium^®^ composites.

Figure 9 shows the load vs. displacement graph of the adhesively bonded Elium^®^ composite laminate. Five samples were tested in this study. The kinks in the curve can be attributed to the difference in the distribution of the adhesive thickness during the adhesion process. This resulted in a loss of adherence due to the difference in crack propagation at some points of the adhesively bonded laminate. The average shear stress value obtained for the ELC_ELC (Elium^®^ composite_ Elium^®^ composite) was 14.2 MPa.

**Figure 10:**
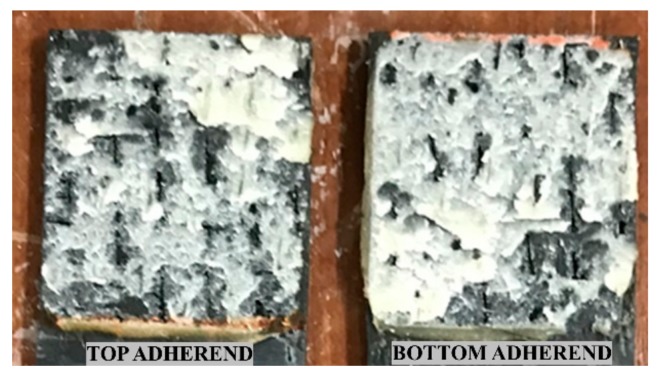
Failure surfaces of adhesively bonded ELC_ELC composites.

Figure 10 shows the fractured interface of the adhesively bonded ELC_ELC joint tested under lap shear testing. Adhesives can be observed on both the adherend surfaces representing a pure cohesive failure. It should be noted that there was no surface preparation carried out on the adherend surfaces. As for the adhesive bond, given that the adhesive was applied to the whole overlap area, the LLS1 and LSS2 values will be similar.

**Figure 11:**
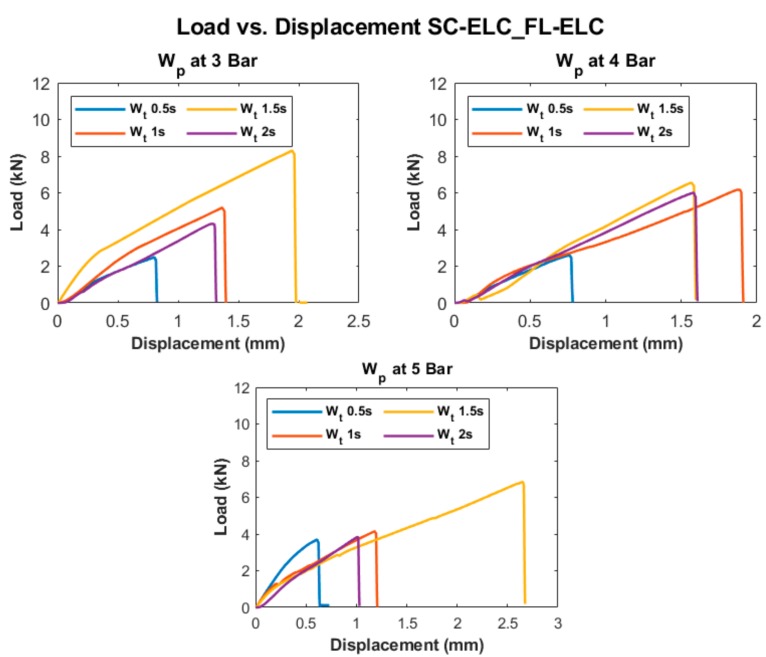
Load vs. displacement curves of SC-ELC_FL-ELC configuration at different welding conditions.

Figure 11 shows the load vs. displacement curves of all welding conditions at three different weld pressures (W_p_), corresponding to four weld times (W_t_) for the SC-ELC_FL-ELC configuration. It should be noted that the graphs show the best representative curve of the average values of the three trials carried out at the same welding condition. A small non-linearity at the start of the curve was observed, which is usually attributed to the backlash in the testing machine and fixture, at around 0.1 mm of displacement. Later, the graphs showed a linear behavior until it reached the maximum load, followed by a drastic load drop, showing the complete failure of the bond in the laminated specimen.

### 4.4. Welding of Elium^®^ Composites with Integrated EDs

**Figure 12:**
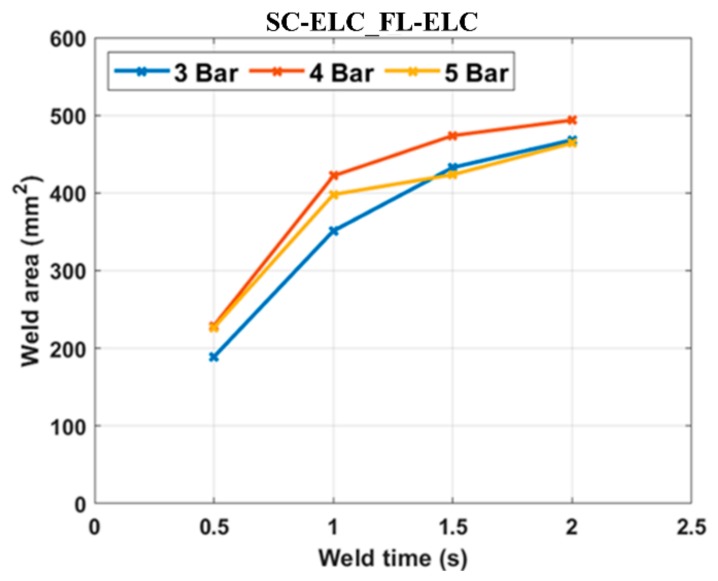
Welded area under all the welding conditions for the SC-ELC_FL-ELC configuration.

As seen in Figure 11, at constant pressure, the maximum load value increased with an increase in the weld time up to an instance after which a further increase in the weld time reduced the load value or the corresponding weld strength of the sample. The weld time after which the load started reducing represents the maximum/optimal weld strength condition corresponding to a specific pressure condition. A similar effect was observed by Wang Tao et. al. where they investigated the ultrasonic welding of CF/PEEK composites at different weld times with and without ED [35]. An increase in weld time led to an increase in energy at the interface. Higher energy at the interface helps in increasing the melting and flowability characteristics of the energy directors and adherends can be welded more efficiently, increasing the weld strength. At higher energy, the matrix of the bottom adherend also melts and significantly adds strength during the fusion bonding. Once an optimal weld strength has been reached, any further increase in weld energy (i.e., weld time) at the interface will reverse the phenomenon and there will be excessive resin flow out of the interface. The insufficient amount of resin results in less deformation and the sample fails easily without taking a significant load. In contrast, at a lower weld time, the amount of energy transferred to the interface is insufficient to melt the resin and fusion bond it to the bottom adherend. From Figure 12, it can be clearly seen that the weld area increased with the increase in weld time for each weld pressure condition. This can be explained as with the increase in weld time, the energy concentration at the interface increases and in turn elevates the temperature at the interface. This results in more melting of the resin and it flows over a larger area. At a higher pressure of 5 bar, the graph shows slightly abnormal behavior. This can be explained by the fact that at this pressure, there is more squeeze out of the resin, which consequently results in a lower weld area compared to 4 bar. At an increased weld time and at 5 bar pressure, this phenomenon starts dominating and leads to a further reduction in the weld area.

**Figure 13:**
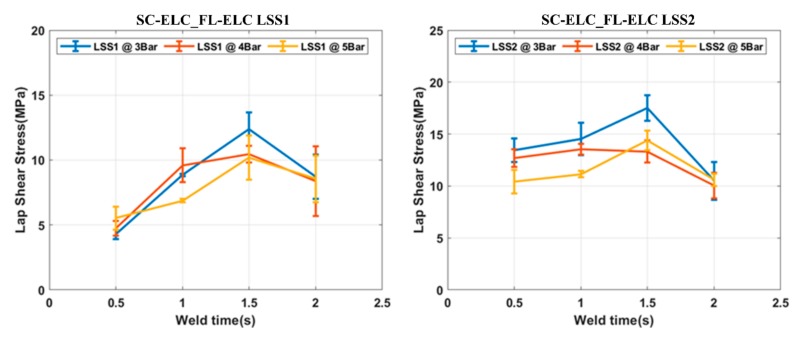
LSS1 and LSS2 graphs for SC-ELC_FL-ELC configuration.

Figure 13 shows the LSS1 and LSS2 values of the specimens at different welding conditions for SC-ELC_FL-ELC. The LSS value showed a similar trend as explained for the load vs. displacement curve. An increase in weld strength was seen with an increase in the weld time at specific pressure, but after the optimal weld time had been reached, it starts to decrease. At higher energy fiber breakage, delamination and other phenomenon were observed. The reason can be explained as the resin being squeezed out of the interface at the higher weld time and pressure, which also damages the adherend, so reducing the weld strength. This phenomenon will be explained further in Section 4.5.

Table 4 shows all the welding combinations carried out for SC-ELC_FL-ELC and its corresponding LSS1 and LSS2 values. As seen from Table 3, the maximum LSS2 value was obtained at the weld time of 1.5 s and 3 bar weld pressure while the minimum LSS2 value was obtained at a 2 s weld time and 3 bar weld pressure. 

### 4.5. Microscopic Investigation and Surface Morphology

**Figure 14:**
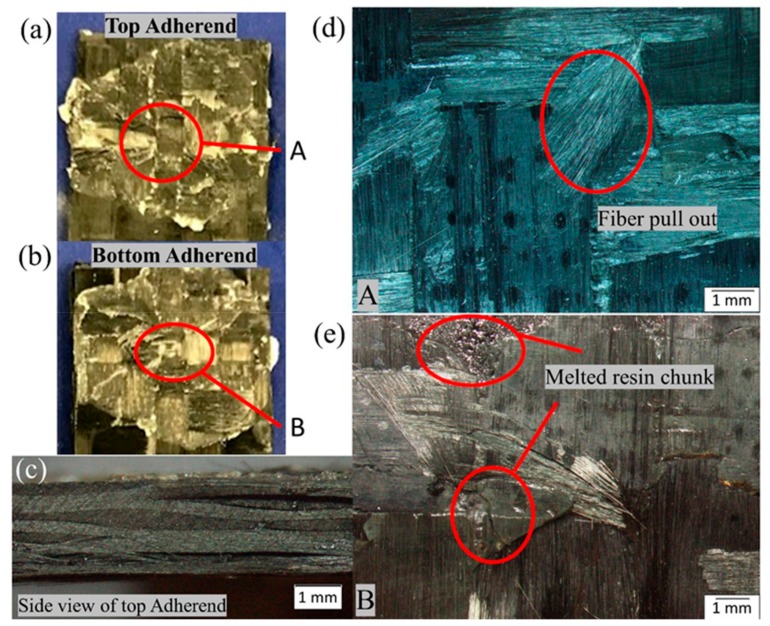
Fracture surfaces of SC-ELC_FL-ELC specimen with maximum LSS value.

**Figure 15:**
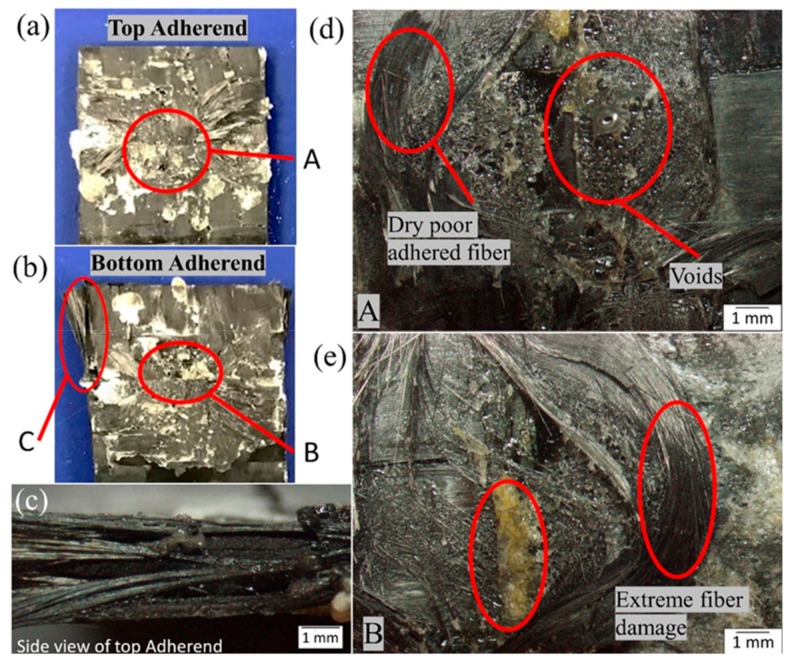
Fracture surface of SC-ELC_FL-ELC specimen with minimum LSS value.

Figure 14 and Figure 15 show the microscopic images of the failed surfaces of both the top and bottom adherend of the SC-ELC_FL-ELC configuration at a maximum and minimum lap shear strength conditions, respectively. As seen in Figure 1, at points A and B, features such as fiber pull out, melted resin, and a chunk of resin can be observed in the bottom adherend. Whereas in Figure 15, at points A and B, the fibers were extensively damaged, and a bundle of fibers had been pulled out, and voids could also be observed. As seen from the side view of Figure 14c, lesser delamination of the top adherend was observed compared to the top adherend shown in Figure 15c, where extensive delamination of the adherend could be observed. Many other bonding features such as fiber imprints, shear cups, matrix deformation, and others that represent an excellent bonding quality were also studied. These features were investigated in-depth by scanning electron microscopy (SEM) observations.

**Figure 16:**
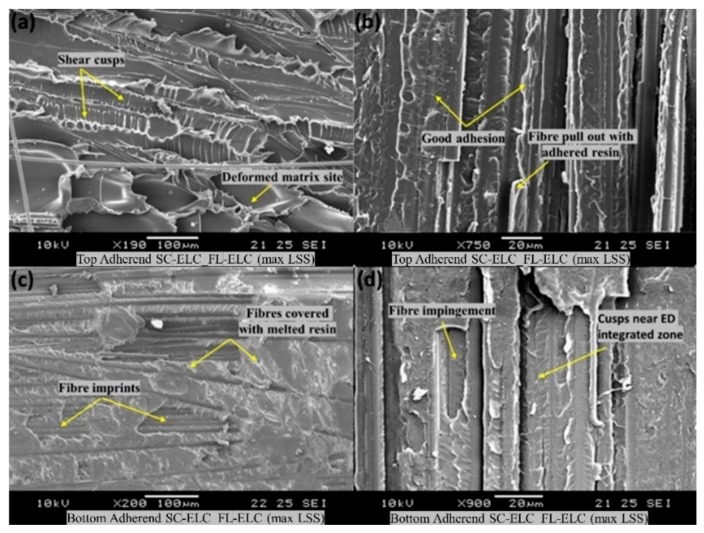
Scanning Electron Microscope (SEM) fractography of SC-ELC_FL-ELC at maximum LSS.

Figure 16 shows the surface morphology of the SC-ELC_FL-ELC composite laminate with the highest lap shear strength of 17.5 ± 1.24 MPa achieved at the following welding parameters: 1.5 s, 3 bar, and 75% amplitude (48 µm). Figure 16a and Figure 15b show the top adherends with shear cusps, plastic deformation sites, good surface adhesion, and the fiber pullout with the adhered resin. All these fractographic features tended to increase the lap shear performance of the welded laminate [65]. The surface morphology study of the bottom adherend as depicted in Figure 16c shows the cohesive failure attribute with fiber rupture and pullout, which was recuperated by the matrix, indicating the damage propagation in the vicinity of the carbon fiber/Elium^®^ interface. Figure 16d shows the clear fiber impingement and the cusps sites near the ED integrated zone. It also shows clear evidence of the melting of the bottom adherend, which is essentially required to achieve excellent weld properties.

**Figure 17:**
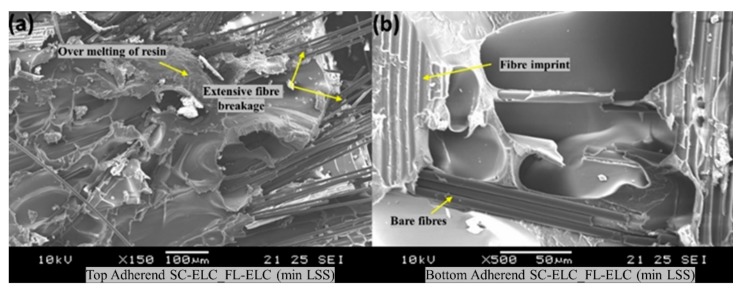
SEM fractography of the SC-ELC_FL-ELC composite for the minimum LSS.

Figure 17 shows the fractography of the SC-ELC_FL-ELC composite laminate with the lowest shear strength of 8.42 ± 1.14 MPa achieved at the welding parameters of 2 s, 3 bar, and 75% amplitude (48 µm). With only a 0.5 s increase in the weld time and keeping the other weld parameters constant, there was significant over melting of the resin and excessive fiber damage occurred on the top adherend (Figure 17a). There were also no signs of cusps near the deformed matrix sites as well as no melting of the resin on the bottom adherend, which was evident by the presence of the bare fibers and cleaner fiber rupture and pullouts.

### 4.6. Comparison of Lap Shear Bonding Strength: Adhesives vs. Welding

**Figure 18:**
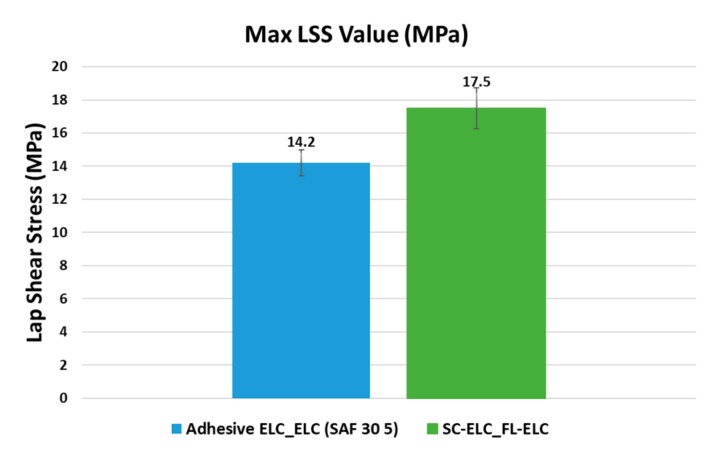
Comparison of the Lap shear stress (LSS) for the laminate joints with adhesively bonded and welded.

Elium^®^ composite was bonded to the Elium^®^ composite by adhesive bonding and by ultrasonic welding. It is to be noted that the comparison for welding was done using the LSS2 value, which is the measure of the weld quality. While comparing the welding results to the adhesive results, it could be clearly seen that the SC-ELC_FL-ELC composite showed a 23% higher LSS value when compared to the adhesive bond strength (SAF 30 5) (Figure 18). Along with the significantly higher bonding strength, the welding time was only 1.5 s as opposed to 10 min of curing time for the adhesive joints.

## 5. Conclusions

Novel carbon Elium^®^ composites with integrated energy directors were successfully manufactured and an experimental study on the influence of different parameters such as weld time, weld pressure, amplitude, ED type, etc. on the weld strength was conducted. The following are the salient findings from the research:

(1) Elium^®^ composites with energy directors can be efficiently welded with the optimized welding parameters of a weld time of 1.5 s and weld pressure of 3 bar. The SC-ELC_FL-ELC welded laminate configuration showed the maximum LSS2 value of 17.5 MPa.
(2) The maximum lap shear strength of the welded laminate (SC-ELC_FL-ELC) was found to be 23.2% higher than the adhesively bonded Elium^®^ laminates.
(3) SEM analysis showed the significant plastic deformation of Elium^®^ resin and the shear cusp formation near the resin-rich sites. These observations were typical of the optimized weld condition and had a direct relationship with strong interfacial bonding.

This research presents an excellent solution to reduce the joining time of Elium^®^ composites in various applications such as automotive, wind turbines, sports, and others with tremendous potential for industrial automation with continuous welding.

## Figures and Tables

**Table 1:** Mechanical, curing, and bonding characteristics of the resin and adhesives used in the current project.

**Matrix**	**Hardener/Initiator**	**Mixing Ratio by Weight**	**Density (g/cm^3^)**	**Viscosity (cP)**	**Shear Strength (MPa)**	**Tg (Glass Transition Temperature) °C**
Elium^®^ 150	Peroxide	100:3	1.2	100 @ 25 °C	22	110
**Adhesive**		**Mixing ratio**	**Open Time (min)**	**Fixture time (min)**	**Lap shear strength (MPa)**	**Curing temperature**
SAF 30 5		1:10	2–3	8	22	RT

**Table 2:** Lap shear testing specification.

ASTM D5868−01 [61]	
Data measured	Force (N), Displacement (mm) or Strain (με)
Mechanical Properties Calculated	Shear strength (MPa)
Specimen dimensions	Length = 101.6 ± 0.2 mm, Width = 25.4 ± 0.1 mm, Thickness = 2 ± 0.01 mm, overlap area = 25.4 × 25.4 mm^2^
Feed rate	13 mm/min
LSS1	Peak load/ total overlap area; Weld efficiency
LSS2	Peak load/ actual welded area; Weld quality

**Table 3:** Design of the experiment for the current research.

Configuration	Weld Time (s)	Weld Pressure (Bar)	Hold Time (s)	Amplitude (%)
SHED_FLAT	0.5, 1, 1.5, 2	3, 4, 5	2	75

**Table 4:** LSS1 and LSS2 test results for the SC-ELC_FL-ELC configuration.

Weld Time (s)	Weld Press (Bar)	LSS2 (MPa)	Std. Dev	LSS1 (MPa)	Std. Dev
0.5	3	13.43	1.14	4.29	0.36
1		14.83	1.13	8.85	0.09
1.5		17.50	1.24	12.37	1.28
2		8.42	1.14	6.92	0.84
0.5	4	12.69	0.86	4.75	0.57
1		13.54	0.51	9.6	1.32
1.5		13.32	1.07	10.45	0.65
2		10.04	1.22	8.36	1.53
0.5	5	10.41	1.14	5.53	0.89
1		11.15	0.32	6.87	0.13
1.5		14.39	0.96	10.19	1.7
2		10.60	0.60	8.55	1.8
